# Challenges in Postoperative Compliance and Follow-Up Among Trauma Patients: A Case Report of a Trans-scaphoid Perilunate Dislocation

**DOI:** 10.7759/cureus.97320

**Published:** 2025-11-20

**Authors:** Paul Bonilla, Anesu K Murambadoro, Chloe Harris, Sergio Rodriguez

**Affiliations:** 1 Orthopedic Surgery, University of Texas Rio Grande Valley School of Medicine, Edinburg, USA; 2 Orthopedic Surgery, Hand and Wrist Institute, DHR (Doctors Hospital at Renaissance) Health, Edinburg, USA

**Keywords:** follow up, fracture-dislocation, hand surgery, noncompliance, trans-scaphoid perilunate fracture dislocation, trauma

## Abstract

The wrist joint is a complex articulation that involves the distal radius, ulna, eight carpal bones (scaphoid, lunate, triquetrum, pisiform, trapezium, trapezoid, capitate, and hamate), and the bases of the metacarpals. Trans-scaphoid perilunate fracture-dislocations are complex wrist injuries, most commonly resulting from high-energy mechanisms involving hyperextension and ulnar deviation. Prompt diagnosis and surgical management are essential in restoring wrist function and stability. This report focuses on the case of a 28-year-old male who sustained a trans-scaphoid perilunate dislocation with multiple associated fractures after an all-terrain vehicle (ATV) rollover accident. Surgical intervention included median nerve decompression, open reduction and internal fixation (ORIF) of the scaphoid, and stabilization of the lunate and associated carpal bones. After receiving initial postoperative care, this patient demonstrated noncompliance with follow-up and rehabilitation, highlighting the challenges that social determinants of health can pose in trauma patient management. This report emphasizes the role of surgical management and postoperative compliance in optimizing patient outcomes.

## Introduction

The proximal row of carpal bones is stabilized by intrinsic and extrinsic ligaments [1]. Trans-scaphoid perilunate fracture-dislocations are caused by high-energy accidents such as falls or road traffic accidents which exert hyperextension and ulnar deviation forces [2,3]. These injuries involve disruption of carpal bones and ligaments, most notably a fracture of the scaphoid combined with lunate dislocation [3-5]. The mechanism involves hyperextension and intracarpal axial rotation. The uncommon nature of this injury stems from the high energy required to fracture the scaphoid and disrupt the overlapping ligaments simultaneously.

Surgical intervention can be required in these injuries to repair fractures and ligaments. The complex nature of wrist anatomy potentiates complications such as stiffness, pain, infections, and post-traumatic arthritis especially if diagnosis or treatment is delayed [6]. Alarmingly, studies indicate that 25% of perilunate injuries are missed which can increase risk of adverse outcomes if not properly managed [7]. Successful recovery depends on rigorous compliance with postoperative care that often includes immobilization, physical therapy, and follow-up visits. Neglected injuries can lead to prolonged periods of instability and abnormal joint mechanics triggering various debilitating conditions [8]. Because sequelae can profoundly affect a patient’s quality of life, it is crucial to monitor adherence to postoperative recommendations.

Noncompliance is particularly concerning in trauma patients, as many of these patients are lost to follow-up, often due to financial constraints. Research indicates that uninsured and Medicaid-insured patients are less likely to initiate or complete recommended follow-up compared to those with private insurance [9-11]. Conversely, higher adherence is consistently associated with improved functional outcomes. These challenges are especially pronounced in the Rio Grande Valley, where socioeconomic disparities and limited access to specialty care hinder continuity of treatment. According to US Census Bureau data, Texas has the highest uninsured rate in the nation at 16.4% and highest percentages being concentrated in the Texas Panhandle, West Texas, and the Lower Rio Grande Valley [12,13]. This case is unique due to the patient’s unique injury, compounded by significant socioeconomic barriers that limited access to timely specialty care and adherence to postoperative rehabilitation. These factors highlight the broader challenges faced by patients in underserved regions such as the Rio Grande Valley, where broader social determinants may influence recovery and long-term functional outcomes including but not limited to financial hardship, transportation barriers, and disparities in healthcare access.

## Case presentation

History and presentation

A 28-year-old Hispanic gentleman presented to the emergency department after an all-terrain vehicle (ATV) rollover accident. Initial assessment at the emergency department revealed a small acute subarachnoid hemorrhage and skull fracture, along with fracture of the left clavicle, wrist, and elbow. The patient was transferred for orthopedic management. The patient presented with complaints of left wrist pain, swelling, and limited range of motion. Physical examination of the left hand showed full flexion and extension of each joint of the digits, with a limited range of motion due to pain at the wrist. The patient reported numbness and tingling over areas consistent with median nerve involvement. Superficial abrasions were noted of volar and ulnar aspects of the left wrist.

Radiograph assessment**

**Figure 1:**
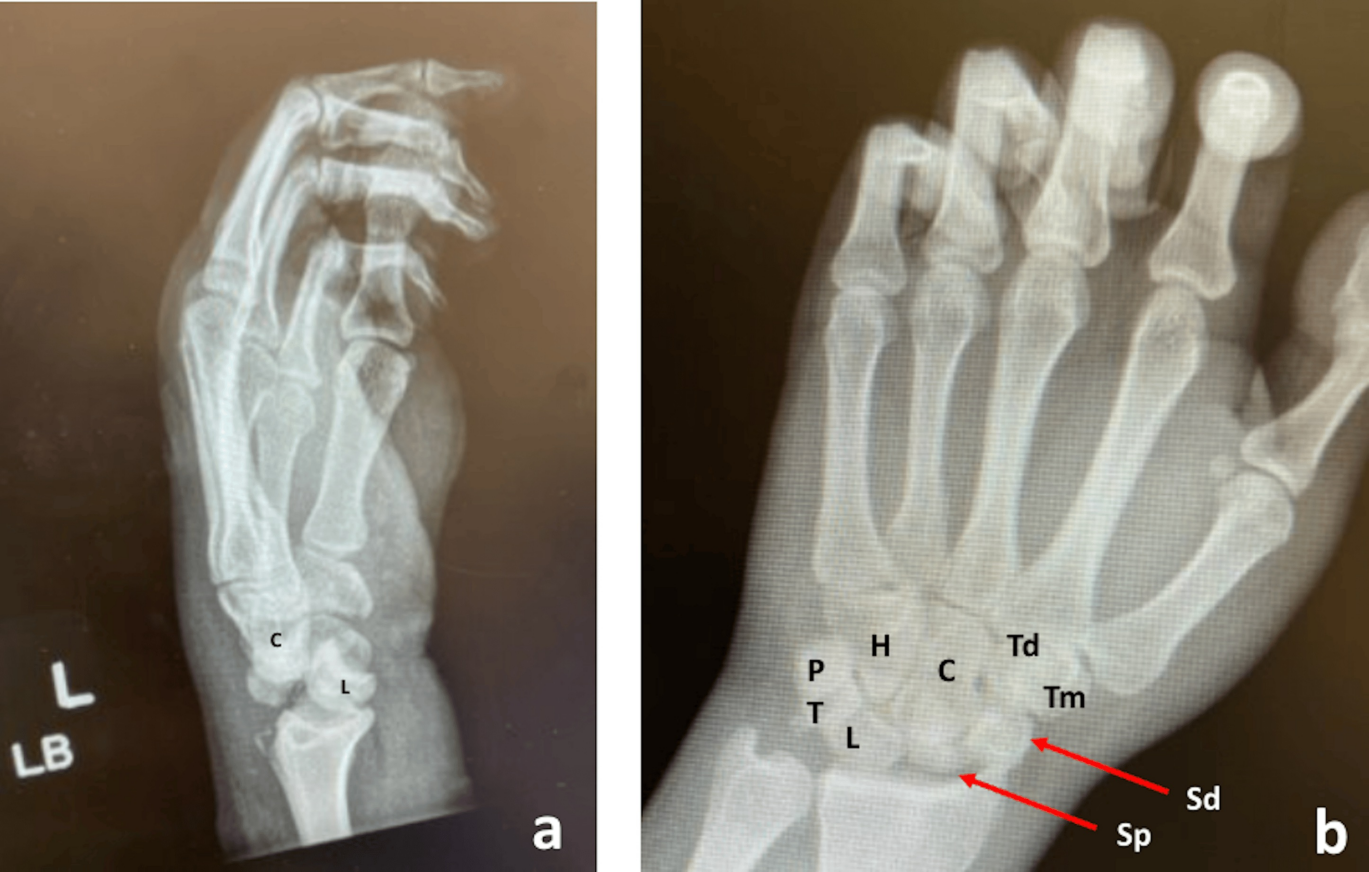
Lateral (a) and posteroanterior (b) view of radiographs showing lunate dislocation and scaphoid fragments. Sd, distal pole of fractured scaphoid; Sp, proximal pole of fractured scaphoid; LB, lateral beam; L, lunate; T, triquetrum; P, pisiform; H, hamate; C, capitate; Td, trapezoid; Tm, trapezium.

An X-ray of the left wrist (anteroposterior, scaphoid, and lateral view) revealed a trans-scaphoid perilunate dislocation with an added fracture of the triquetrum (Figures 1a, 1b).

**Figure 2:**
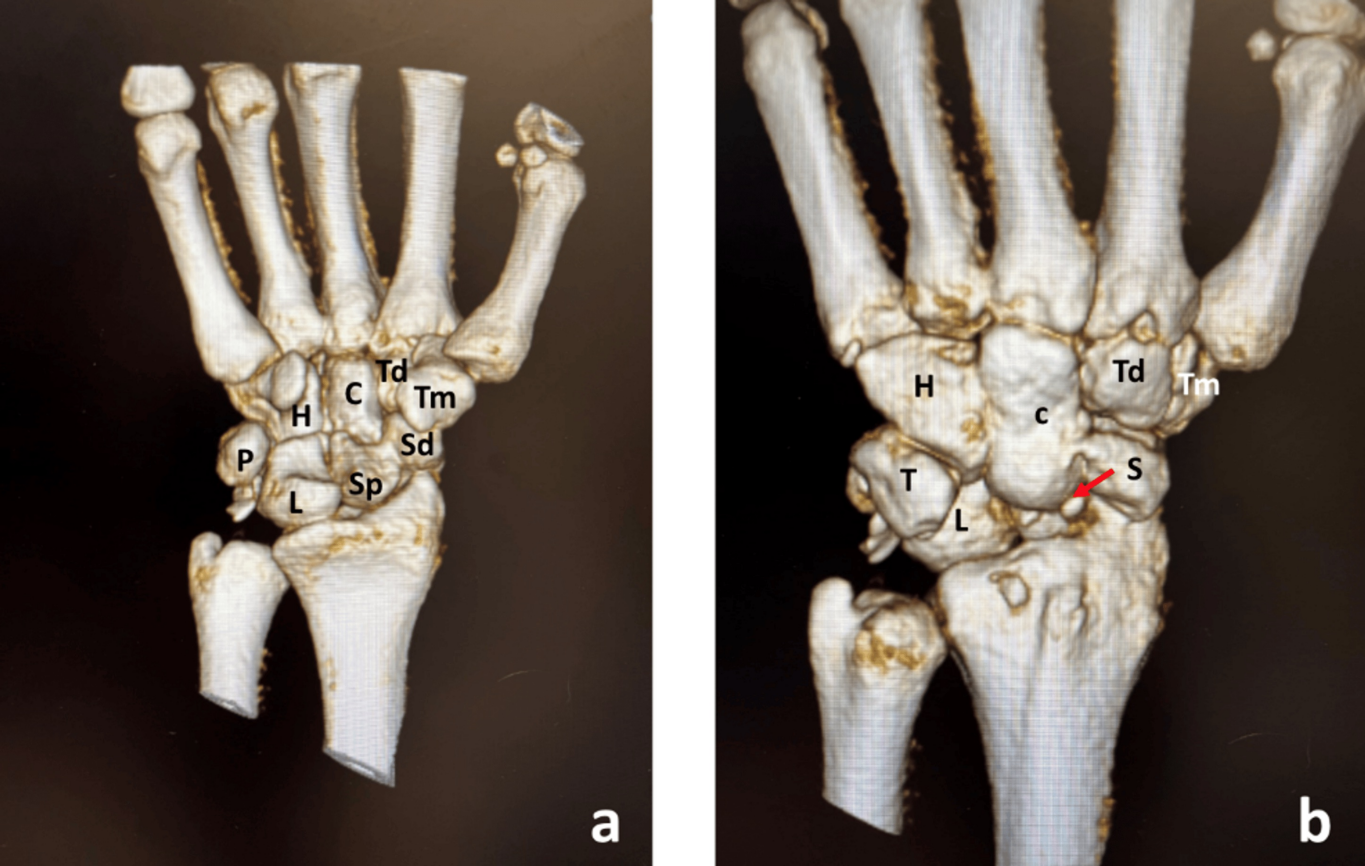
3D CT posteroanterior (a) and anteroposterior view (b). Arrow indicates fractured proximal pole of scaphoid. Sd, distal pole of fractured scaphoid; Sp, proximal pole of fractured scaphoid; L, lunate; T, triquetrum; P, pisiform; H, hamate; C, capitate; Td, trapezoid; Tm, trapezium.

CT scan with reconstruction of the left wrist revealed trans-scaphoid perilunate dislocation. Scaphoid waist fracture is displaced and overlapping. Small fracture fragments are noted at the fracture site. There is a small, displaced fracture fragment arising from the dorsal aspect of the lunate and a displaced fracture of the inferior aspect of the triquetrum. A small fracture fragment at the dorsal aspect of the distal radius is of uncertain origin (Figures 2a, 2b).

Management

Surgery began with decompression of the median nerve at the wrist on 08-11. A longitudinal incision was made along the radial border of the ring finger over the transverse carpal ligament. After skin incision with a #15 blade, subcutaneous tissues were bluntly dissected with tenotomy scissors. The palmar aponeurosis was identified and divided, followed by careful dissection to the transverse carpal ligament, which was incised from distal to proximal while protecting the vascular arch. A substantial hematoma was encountered within the carpal tunnel, exerting pressure on the intact median nerve. The hematoma was evacuated, the tendon sheaths were opened, and the distal fibers of the antebrachial fascia were released. The thenar motor branch was intact, and no masses were present. The wound was irrigated with antibiotic-impregnated saline and temporarily covered with moist gauze.

A dorsal approach was used for the left wrist. A longitudinal dorsal incision revealed a significant soft tissue hematoma, which was evacuated. The sensory branches of the radial nerve were preserved. The third dorsal extensor compartment was opened, and the extensor pollicis longus tendon was radialized. The dorsal extensor retinaculum between the second and fourth compartments was divided, allowing retraction of the tendons and exposure of the posterior interosseous nerve, which was resected over a 1.5 cm segment to aid in postoperative pain prevention. The dorsal wrist capsule was noted to be avulsed from the distal radius.

An inverted-T capsulotomy was performed for exposure. The distal pole of the scaphoid and capitate was visualized, with the capitate intact. The triquetrum fracture was identified, with the proximal pole of the scaphoid and fractured triquetral fragment remaining attached to the lunate via intact scapholunate and lunotriquetral ligaments.

The Tavernier’s maneuver was used to reduce the perilunate dislocation under fluoroscopic guidance. The scaphoid waist fracture was then reduced and stabilized with an AcuTrak (Acumed, LLC., Hillsboro, Oregon, United States) mini headless compression screw (24 mm), placed over a guidewire inserted along the longitudinal scaphoid axis. Fluoroscopy confirmed optimal screw position and fracture reduction.

**Figure 3:**
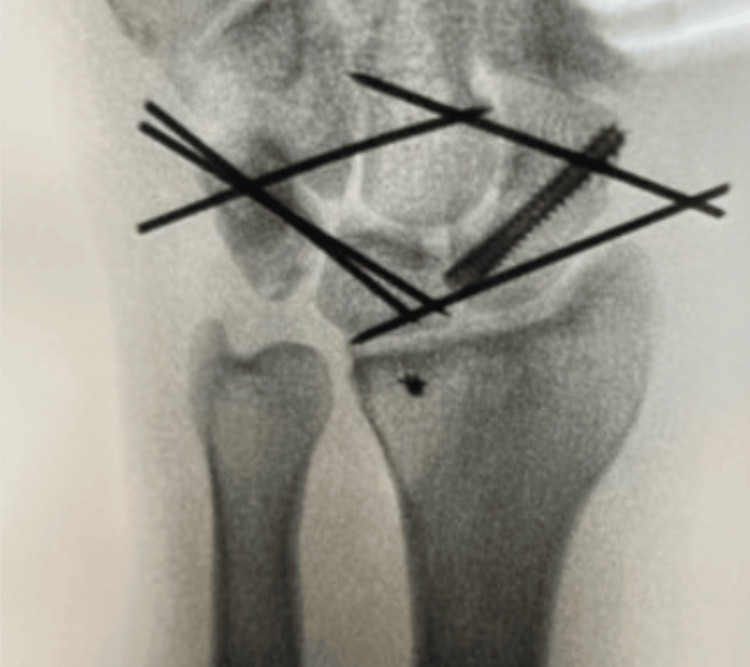
Posteroanterior view of radiograph showing postoperative surgical fixation with a scaphoid screw, pins, and mini anchor.

Stabilization of the lunate and triquetrum was achieved with two 0.045-inch K-wires, while maintaining volar pressure on the capitate. Additional K-wires were placed from the triquetrum to capitate, distal scaphoid to capitate, and proximal scaphoid to lunate, creating a diamond-shaped configuration for stability (Figure 3). All pin placement was guided by intermittent fluoroscopy.

The dorsal capsule was reattached to the distal radius with a mini anchor and FiberWire (Arthrex, Inc., Naples, Florida, United States) sutures. All wounds were copiously irrigated with antibiotic saline. Final intraoperative radiographs confirmed anatomic reduction and proper hardware placement. The dorsal extensor compartment was repaired, leaving the extensor pollicis longus radialized. Hemostasis was secured after tourniquet release, and all incisions were closed with sutures and staples.

For postoperative analgesia, 30 cc of 0.75% plain Marcaine was infiltrated. Bacitracin, Xeroform, and a bulky sterile dressing were applied. Immobilization was achieved with a well-padded thumb spica short-arm splint, supplemented with a posterior long-arm splint for concurrent elbow fracture protection, and a sling for clavicle immobilization.

The patient was transferred to the post-anesthesia care unit in stable condition and later discharged home. Planned follow-up included suture and staple removal at two weeks, initiation of rehabilitation, and continued orthopedic and trauma service oversight for concurrent injuries, including clavicle fracture, elbow fracture, skull fracture, and subarachnoid hemorrhage.

Follow-Up

**Figure 4:**
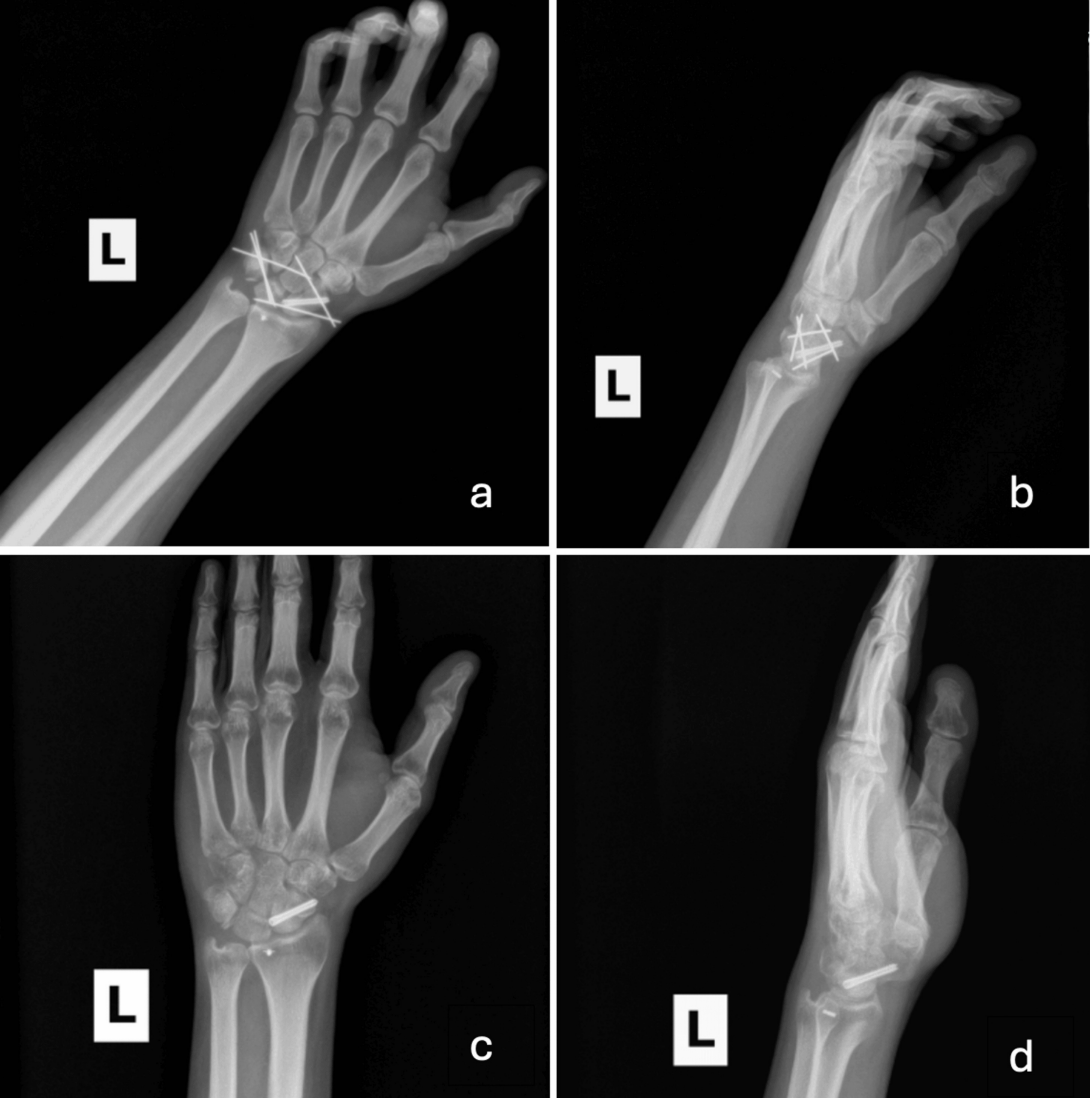
Posteroanterior (a, c) and lateral (b, d) view of pre- and post-hardware removal radiographs.

The patient demonstrated poor compliance with recommended care and missed his scheduled clinic visit and hand therapy appointment two and a half weeks postoperative, as well as his follow-up with the orthopedic trauma service for concomitant clavicle and radial head fractures. The patient then showed up unannounced two days after his scheduled visit and demanded to be seen. On examination, he reported joint stiffness and decreased range of motion in the left wrist. Staples were removed without complication, and the patient was counseled extensively on the importance of adherence to postoperative restrictions, including strict avoidance of lifting, pushing, pulling, climbing, carrying, or engaging in any physical activity with the affected hand. The patient subsequently returned eight week postoperative for preoperative evaluation and counseling for hardware removal, which was performed uneventfully the next day (Figures 4a-4d). Ten-week postoperative follow-up demonstrated wrist range of motion at 25° extension, 15° flexion, full pronation and supination, 12° radial deviation, and 25° ulnar deviation.

Continued rehabilitation was advised, with plans for reassessment and radiographic evaluation in six weeks. However, the patient failed to attend the scheduled four-month postoperative appointment.

Timeline of events summary

Injury (08-11) → Surgery (08-11) → two-week postoperative for suture and staple removal: missed visit (08-29) → Unannounced visit (08-31) → Preoperative visit for hardware removal: attended (10-05) → Hardware removal surgery (10-06) → 10-week postoperative evaluation: attended (10-26) → four-month postoperative evaluation: missed visit (12-12).

## Discussion

Trans-scaphoid perilunate fracture-dislocations represent a rare, high-energy wrist injury that requires management of both osseous and ligamentous disruption. Surgical management in this case required staged release and decompression of the median nerve to address acute carpal tunnel syndrome caused by hematoma, followed by dorsal wrist exposure and reduction of the fracture-dislocation. Fixation was achieved using a combination of a headless compression screw and strategically placed K-wires in a diamond configuration to stabilize the lunate and associated fractures. The dorsal capsule was repaired to restore stability, and meticulous intraoperative fluoroscopy confirmed anatomical reduction. This approach reflects the principle that both osseous and ligamentous structures must be addressed in perilunate injuries to achieve durable functional outcomes [14].

Despite technically successful surgical reconstruction, the patient’s recovery was hindered by noncompliance with postoperative care. He missed multiple clinic visits, hand therapy appointments, and follow-ups with other treating specialists. This nonadherence likely increased the risk of joint stiffness, impaired rehabilitation progress, and suboptimal functional recovery. Optimal results cannot be attained through surgical intervention alone. Strict postoperative care and rehabilitation adherence is necessary to ensure proper healing and recovery. Noncompliance not only jeopardizes patient improvement but also strains the healthcare system through postoperative complications, revisions, or the need for extended rehabilitation. Studies show that scheduling follow-up appointments alone does little to improve return rates, and patients lost to follow-up often re-enter the system only when complications arise [15]. This underscores the need for proactive, multifaceted strategies to maintain postoperative engagement.

The influence of social determinants of health (SDoH) in this context cannot be overstated. Factors such as socioeconomic status, transportation limitations, competing work or family demands, and health literacy directly impact a patient’s ability to adhere to treatment. Studies have shown that improving adherence requires more than appointment scheduling; it benefits from building strong patient-physician relationships, establishing shared health goals, and incorporating early involvement of social services [4,16,17]. In rural and medically underserved areas, these barriers are magnified. The Rio Grande Valley, which has one of the highest uninsured rates in the United States and is classified as rural by the U.S. Census Bureau, exemplifies such a setting. Prior research in rural populations has identified disinterest, competing demands, and insufficient system support as major barriers to attendance [17,18]. Physicians must recognize potential barriers to adherence before discharge to tailor a postoperative plan that accommodates the patient’s needs.

## Conclusions

This case reinforces the need for a multidisciplinary approach to postoperative care in high-risk populations. Incorporating patient education programs, coordinated social work support, and telehealth follow-up may help mitigate barriers and improve adherence. Ultimately, achieving long-term functional recovery in trans-scaphoid perilunate fracture-dislocations requires both surgical precision and deliberate strategies to address the social and logistical challenges that threaten continuity of care.

## References

[1] Eschweiler J, Li J, Quack V, Rath B, Baroncini A, Hildebrand F, Migliorini F (2022). Anatomy, biomechanics, and loads of the wrist joint. Life (Basel).

[2] Stanbury SJ, Elfar JC (2011). Perilunate dislocation and perilunate fracture-dislocation. J Am Acad Orthop Surg.

[3] Ambulgekar RK, Masne PS, Jadhav A (2006). Trans-scaphoid perilunate fracture dislocation managed with open reduction: a case report. J Orthop Case Rep.

[4] Mayfield JK, Johnson RP, Kilcoyne RK (1980). Carpal dislocations: pathomechanics and progressive perilunar instability. J Hand Surg Am.

[5] Aslani H, Bazavar MR, Sadighi A, Tabrizi A, Elmi A (2016). Trans-scaphoid perilunate fracture dislocation; a technical note. Bull Emerg Trauma.

[6] Divecha HM, Clarke JV, Barnes SJ (2011). Established non-union of an operatively managed trans-scaphoid perilunate fracture dislocation progressing to spontaneous union. J Orthop Traumatol.

[7] Palmer AK, Werner FW, Murphy D, Glisson R (1985). Functional wrist motion: a biomechanical study. J Hand Surg Am.

[8] Jerome JT (2025). Neglected perilunate injuries: management strategies and long-term consequences: a narrative review. J Musculoskelet Surg Res.

[9] Gupta SK, Troyer LD, Si Z, Gieg SD, Leary EV (2024). Race, income, and insurance status are associated with increased time to initial outpatient evaluation of fracture patients. J Pediatr Soc North Am.

[10] Bhashyam AR, Challa ST, Thomas H, Rodriguez EK, Weaver MJ (2023). Clinic follow-up of orthopaedic trauma patients during and after the post-surgical global period: a retrospective cohort study. BMC Musculoskelet Disord.

[11] Bender M, Jain N, Giron A, Harder J, Rounds A, Mackay B (2024). Factors influencing compliance to follow-up visits in orthopaedic surgery. J Am Acad Orthop Surg Glob Res Rev.

[12] (2025). Texas 2036: Where are the uninsured located in Texas? Part 1: The county view. https://texas2036.org/posts/where-are-the-uninsured-located-in-texas-part-1-tx-county-view/.

[13] (2025). U.S. Census Bureau: Health. https://data.census.gov/profile/Texas.

[14] Kara A, Celik H, Seker A, Kilinc E, Camur S, Uzun M (2015). Surgical treatment of dorsal perilunate fracture-dislocations and prognostic factors. Int J Surg.

[15] Haider AH, Figura RH, Ladha K (2014). Can we decrease the number of trauma patients 'missing in action'? A prospective pilot intervention to improve trauma patient compliance with outpatient follow-up at an urban Level I trauma center. Am Surg.

[16] Perche PO, Singh R, Cook MK, Kelly KA, Balogh EA, Richardson I, Feldman SR (2023). The patient-physician relationship and adherence: observations from a clinical study. J Drugs Dermatol.

[17] Aaland MO, Marose K, Zhu TH (2012). The lost to trauma patient follow-up: a system or patient problem. J Trauma Acute Care Surg.

[18] Chapman KA, Machado SS, van der Merwe K, Bryson A, Smith D (2022). Exploring primary care non-attendance: a study of low-income patients. J Prim Care Community Health.

